# Managing robotics in the generic pharmaceutical arena

**DOI:** 10.1155/S1463924692000099

**Published:** 1992

**Authors:** Marianne Scheffler

**Affiliations:** Danbury Pharmacal, Inc 12 Stoneleigh Avenue Carmel New York 10512 USA

## Abstract

Robotics was introduced by Danbury Pharmacal, Inc. in 1987 in order to improve laboratory throughput for several new products. The author uses Danbury Pharmacal's experience to give an overview of various issues, such as acceptance by senior management and chemists, political confrontation, validation and product throughput.


Journal of Automatic Chemistry, Vol. 14, No. 2 (March–April 1992), pp. 37–41

### Managing robotics in the generic pharmaceutical arena

**Marianne Scheffler**

*Danbury Pharmacal, Inc., 12 Stoneleigh Avenue, Carmel, New York 10512, USA*

*Robotics was introduced by Danbury Pharmacal, Inc. in 1987 in order to improve laboratory throughput for several new products. The author uses Danbury Pharmacal's experience to give an overview of various issues, such as acceptance by senior management and chemists, political confrontation, validation and product throughput.*

### Introduction

The pharmaceutical industry, both generic (multisource), as well as PMA ('Brand') firms have a primary obligation to provide to the user (patient) finished dosage forms which meet all mandated standards for identity, purity, strength, and quality. A key difference between a generic pharmaceutical or multisource company and a major PMA firm are the larger number and greater variety of products manufactured by the generic firm. The pharmaceutical industry continues to be challenged with stricter government regulations, which has placed increasing demands on firms for better and faster analytical testing capabilities. Pressure to meet production schedules without compromising product quality often becomes the driving force to improve laboratory productivity. Improving laboratory productivity is complicated by shorter product life-cycles, coupled with the continuing changes in specifications and test methods made by the FDA and the USP/BP/PF etc. Each time a change in requirement occurs, a whole battery of time-consuming activities ensues: validation of modified test methods, verification of compliance to the new specifications, updating laboratory documentation etc.

Increased productivity can often be achieved from the existing laboratory staff by allowing individuals to concentrate on the problem areas, while the laboratory equipment performs the repetitive work. These include the routine use of autosamplers for titrators, HPLC systems, and GC systems, automated dissolution apparatus, etc. The author's company, like most firms, has also tried to achieve this goal by introducing robotics. A Zymark Pytechnology II robot was purchased in 1987 by Danbury Pharmacal. The robot pysections were developed to perform content uniformity analysis with detection by a Diode Array UV/Vis spectrophotometer. Content uniformity testing is one of the major time-consuming and repetitive tasks for the chemist.

The development and continued success of Danbury Pharmacal's robotics efforts resulted from a continuously increasing work-load, coupled with the presence of 'technical champions' for automation. This paper examines some key issues that the organization's automation effort raised.

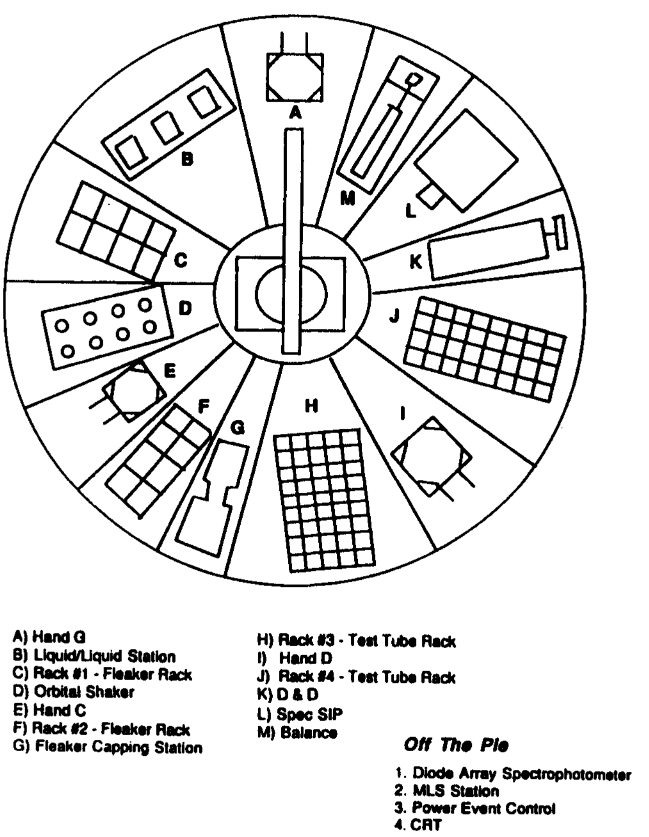

*Figure 1. Zymark Pytechnology II robot.*

### Commitment

The most important factor in the success of any laboratory robotics system is support and commitment by management. Unrealistic commitments are often made to managers about the advantages and productivity of robotics systems. This is particularly true when a robotic system is introduced as a research tool rather than as a turn-key acquisition for a particular assay. Soon after the initial acquisition of the robot at Danbury Pharmacal, the robotic staff chemist assigned to validate and automate methods on the robot became urgently needed for other tasks. When significant delays, due to unpredicted problems, occur during the development of a robotic assay intended to replace a labour-intensive manual assay, management's enthusiasm rapidly diminishes. At this point, purely technical issues become political issues. Questions arose such as 'Why is the robot not producing', 'When will it be on-line?', and 'Why have we spent all this money for the robot to collect dust?'. However, clear thinking about the best type of application for the robot often leads to identifying the proper types of methodologies to automate. Such was the case with the author's company and the robot was put back to effective use in the laboratory.

© Zymark Corporation 1992

### Staffing and location

### *Staffing*

Staffing was a real problem in implementation: whether to have a computer programmer or a chemist. It was decided that successful automation of a sample preparation and analysis would require close involvement of an individual in the scientific discipline. In chemistry, for example, many of the failures of automation can be traced to situations where engineers or computer programmers were expected to make decisions and judgments about chemical procedures. Of course, a knowledge of automation techniques was important too. However, it appears to be easier for a chemist to understand the automation needs than it is for an engineer or a computer programmer to understand all the nuances of chemistry.

### *Location*

The robot was located between the quality control (QC) and research and development (R & D) laboratories. The room was not the most suitable for making repairs to the robot, but it is operational. A compressed air line was installed to handle the liquid/liquid and balance pysections. Laboratory reagents are prepared in either the QC or R & D laboratory and brought into the robotic room for the robot to handle. The ideal situation would be to design the laboratory space prior to the installation of the robotic system. For instance, all services, such as water, air, vacuum, temperature control, '360' access, etc., should be considered and instituted as part of the laboratory design. Ample room should also be provided for any future expansion.

### Approaching automation

To implement robotics or not? The key issue in selecting the appropriate pysections for the robot is to first consider what product(s) might be suitable for automation. Issues such as how often is the product being manufactured, and, how complex is the manual methods, should all be taken into consideration. A careful review must be conducted to search for a robotics application so that appropriate pysections can also be identified and purchased. The author's company's robot was purchased, instead, with a process in mind, content uniformity, not a product. However, not all products would be amenable to automation on this robot due to the limitations of the analysis detection by a UV/Vis spectrophotometer. Indeed, many high volume products are analysed by HPLC. The robotic staff chemists have identified four product lines that can be considered for automation. In summary, management must recognize that the best applications which can benefit may not be attainable with the equipment initially purchased, and to understand the reasons why implementation is not always easy, even if the application is appropriate.

Another type of political issue that often accompanies the installation of robotics in a busy QC laboratory environment is 'job security'. Many chemists still feel that robotics will replace their jobs. Others say that the robot could not be as precise as the chemist. One way of changing this thinking is to show that the chemists can now perform the other tasks required to complete the analysis work for the product, or concentrate on other projects. Another approach is to ask whether the chemists would prefer to perform manual injections on an HPLC system or use an autosampler—the majority of chemists will choose the autosampler. With time, a full and complete acceptance of robotics will be inevitable.

Currently, only one product line has been released for routine testing by the present robot, and several other products are going through the final stages of automation validation. Other technologies are concurrently being investigated by the robotic staff chemists to aid the automation effort, to reduce chemist analysis time, and still manage the large volume of products being manufactured. The issues of automating other methods are also being considered and reviewed.

Robotics is not the answer to all laboratory problems. An automated method could often have a longer robotic analysis time than a chemist performing the task manually. However, it must be taken into consideration that robots do not take lunch breaks, coffee breaks, vacation, or chat with their fellow robots. They can operate 24 hours a day, seven days a week, 365 days a year.

By automating an analytical method, the human error factor has been reduced. Automation eliminates possible errors, such as incorrect dilutions, weighings, miscalculations, and transcription errors. The reliability of any robotic or automated method ultimately is a direct reflection on the creative chemist who automated the methods and successfully validated the process.

### Validation

Validation of the robot is accomplished in two stages. Stage one involves the validation of the bench-top configuration. It is important to remember that the specific application for the pysection requires thorough examination for validation considerations. The bench-top validation can be elaborate and complex, or a simplistic approach can be implemented. We have given this decision to the robotic staff chemist performing the validation. The bench-top validation process provides the robotic staff chemist experience in all phases of the robotic operation required for programming, and allows them to become familiar with the Pytechnology II robot. The robot's pysections are as follows:

(2) 16 × 100 mm test tube racks

(1) Fleaker Capping Station

(1) Orbital Shaker

(1) Spec Sip Station

(1) Dilute and Dissolve

(1) Hand D

(1) 0·2 ml–1·0 ml Hand G

(1) Hewlett-Packard Diode UV/Vis Spectrophotometer

(2) 250 ml Fleaker Cap Racks
(1) Liquid/Liquid Pysection
(1) Mettler Balance
(1) Master Lab Station (MLS)
(1) Power & Event Controller
(1) Hand C.

Stage two involves creating the automated method and validating the automated process; the two validation stages need to be further explained.

*Benchtop configuration validation*

The benchtop validation consisted of grouping various robotic procedures to validate several pysections simultaneously. Some of the benchtop programs are also utilized during the validation process for automating a method. For example, a typical benchtop validation program consists of utilizing the test-tube racks, dilute and dissolve, analytical balance, and the MLS station. Grouping several robotic functions gives the ability to assure, and monitor simultaneously, the capability of the sample numbering sequence, grasping and placement of an appropriate test-tube, and monitoring the accuracy of dispensing a liquid in correlation to the weight received on the analytical balance. In this case, water was used as the solvent to conduct the benchtop validation, but this program can also be employed for any other solvent system that is desired. An example of the data collected is displayed in table 1. Benchtop validation is performed upon installation, or if the unit must be disassembled and moved to a new location, or if the benchtop configuration changes due to the installation of a new pysection, upgrades, and/or major repairs.

*Validating the automated method*

After successfully validating and documenting the benchtop configuration, the creation of a program for automating a manual method begins. We approached this by simulating robotically what the chemist would perform manually with minimal process modifications. A final and important step before routine use of any automated sample preparation system is to validate the integrity of the method. The object of the validation test is to prove that there are no procedural errors in the automated sample preparation method. The obvious analysis is to verify that the samples at the end of the process are in a form suitable for their intended use. The less obvious factor is the need to be sure that there are no significant variables that affect the samples that may not be controlled.

Stage two now involves validating the automated method. The automated method was adapted from an established manual method. Linearity, accuracy, precision, recovery, and equivalency of the automated method were compared with the manual method.

During the validation process, difficulty was found recovering the analyte from the product excipients. To correct the recovery problem, a slight modification of the sample extraction preparation was implemented. It was noticed, during other product validations, that the limiting factor in automating the method was due to the orbital shaker. This was concluded by breaking down the various robotic steps and carrying on the remainder of the process manually to identify the probable cause. The orbital shaker dissolves the solid dosage form, but it does not use the same action as a mechanical shaker or sonicator. However, there are variations that can be implemented, and documented, to the sample preparation procedure that can alleviate these limitations.

*Table 1. MLS Syringe B Validation.*

MLS syringe B dispensing 2·0 ml of water

| Sample number | Sample weight | Corrected volume for density |
|---|---|---|
| 1 | 1·9940 g | 2·002 ml |
| 2 | 1·9936 g | 2·002 ml |
| 3 | 1·9932 g | 2·001 ml |
| 4 | 1·9933 g | 2·001 ml |
| 5 | 1·9943 g | 2·002 ml |

| Mean volume | SD | % RSD | Accuracy |
|---|---|---|---|
| 2·0017 | 0·0004 | 0·0209 | 0·0843 |

MLS syringe B dispensing 5·0 ml of water

| Sample number | Sample weight | Corrected volume for density |
|---|---|---|
| 6 | 4·9963 g | 5·016 ml |
| 7 | 4·9957 g | 5·016 ml |
| 8 | 4·9960 g | 5·016 ml |
| 9 | 4·9957 g | 5·016 ml |
| 10 | 4·9955 g | 5·016 ml |

| Mean volume | SD | % RSD | Accuracy |
|---|---|---|---|
| 5·0159 | 0·0003 | 0·0056 | 0·3181 |

MLS syringe B dispensing 7·0 ml of water

| Sample number | Sample weight | Corrected volume for density |
|---|---|---|
| 11 | 6·9992 g | 7·027 ml |
| 12 | 6·9984 g | 7·027 ml |
| 13 | 6·9977 g | 7·026 ml |
| 14 | 6·9978 g | 7·026 ml |
| 15 | 6·9976 g | 7·026 ml |

| Mean volume | SD | % RSD | Accuracy |
|---|---|---|---|
| 7·0262 | 0·0006 | 0·0085 | 0·3749 |

MLS syringe B dispensing 10·0 ml of water

| Sample number | Sample weight | Corrected volume for density |
|---|---|---|
| 16 | 10·0086 g | 10·049 ml |
| 17 | 10·0089 g | 10·049 ml |
| 18 | 10·0091 g | 10·049 ml |
| 19 | 10·0088 g | 10·049 ml |
| 20 | 10·0087 g | 10·049 ml |

| Mean volume | SD | % RSD | Accuracy |
|---|---|---|---|
| 10·0490 | 0·0002 | 0·0019 | 0·4902 |

Performing the equivalency portion of the validation for the manual methods versus the automated method, provided very encouraging data. Five randomly manufactured lots were analysed for the two potencies. The products were analysed for content uniformity and assay by both methods to determine the comparability of the methods. Again, content uniformity is performed on 10 individual dosage units for an existing batch, and two sample weights are taken from a composite blend of 20 tablets for the assay. Zymark's robot proved to be

*Table 2. Analytical data for content uniformity/assay.*

| | | | | |
|---|---|---|---|---|
| Standard number | Std. wgt (gm) | Standard abs. | | |
| 1 | 0·0532 | 0·5434 | | |
| Sample number | Sample wgt (gm) | Sample abs. | Conc. (mg/tab) | % label |
| 1 | 0·5055 | 0·5569 | 98·140 | 98·14 |
| 2 | 0·5120 | 0·5715 | 100·716 | 100·72 |
| 3 | 0·5080 | 0·5806 | 102·318 | 102·32 |
| 4 | 0·5064 | 0·5859 | 103·240 | 103·24 |
| 5 | 0·5108 | 0·5881 | 103·641 | 103·64 |
| Standard number | Std. wgt (gm) | Standard abs. | | |
| 2 | 0·0532 | 0·5595 | | |
| Sample number | Sample wgt (gm) | Sample abs. | Conc. (mg/tab) | % label |
| 6 | 0·5090 | 0·5747 | 101·270 | 101·27 |
| 7 | 0·5095 | 0·5772 | 101·710 | 101·71 |
| 8 | 0·5146 | 0·5789 | 102·006 | 102·01 |
| 9 | 0·5086 | 0·5766 | 101·606 | 101·61 |
| 10 | 0·5114 | 0·5774 | 101·754 | 101·75 |
| Standard number | Std. wgt (gm) | Standard abs. | | |
| 3 | 0·0532 | 0·5542 | | |
| Calc. tab. mean. wt | SD | % RSD | Avg. (% label) | |
| 0·5096 | 1·506 | 1·482 | 101·64 | |
| Assay number | Sample wgt (gm) | Sample abs. | Conc. (mg/tab) | % label |
| 1 | 0·5150 | 0·5719 | 99·904 | 99·90 |
| 2 | 0·5160 | 0·5773 | 100·646 | 100·65 |
| Standard number | Std. wgt (gm) | Standard abs. | | |
| 4 | 0·0532 | 0·5552 | | |
| Avg. tab. wgt (gm) | Avg. conc. | Avg. (% label) | | |
| 0·5127 | 100·275 | 100·27 | | |

Chemist:
Date: July 16, 1991

Product:
Status: Coated
Potency: 100 mg
Lot number:

Book number:
Page number(s)

Standard lot number: 84
Standard purity: 0·9980

accurate and precise: statistically there was no significant difference noted between the data collected from the manual method versus the robotic method. The critical parameter indicated that there was a 95% confidence level performing a paired t-test.

*Documentation*

Documentation should be prepared as the system program parameters are being developed, and not as a separate entity at the end of a project. The objective of the documentation is to record all experiments that occurred during the validation process. A final validation report should document the events and demonstrate the results obtained. The documentation process also allows a new person to operate and maintain the system.

### Robotic analysis

Danbury Pharmacal has released one product line using the robotic results obtained for both assay and content uniformity. This product is in the form of a film-coated tablet; after the laboratory submits the results for releasing the core product, it is then film-coated by the manufacturing department, and another random sampling of the same lot is sent to the laboratory for analysis. The chemist's involvement in the robotic process is approximately 45 minutes. This time is spent replenishing disposable items such as test-tubes, pipette tips and filters, placing the samples and standards in the appropriate positions, and preparing the sample extraction preparation solution. Finally, the chemist documents the data in the laboratory notebook.

Full data reports are calculated and generated by the robot so the chemist can concentrate on interpreting the data. An example of a report generated by the robot is given in table 2. All pertinent reporting information for the robot has been programmed to be presented on a single piece of paper. This includes the weights, absorbance readings, percentage of label results, statistics, operator, date, and the laboratory notebook volume and page where the results can be located.

### Multi-task events

As was stated previously, the robot is releasing the product for content uniformity and assay for this particular product line. To properly manage the workload for the robot, overnight release testing is performed. A product is placed on the robot in the late afternoon and so the robot can operate overnight unattended. The chemist retrieves the final results the following morning and reviews the data to ensure that no system problems have occurred.

Danbury Pharmacal's robot is utilized during the day to validate other products. It is essential to take into consideration, while automating other products on the same robot, the possibility of contamination. Controlling the number of different methods dedicated to the robotic system would minimize, if not reduce, the possibility of contamination. Typically, analyses that have like systems for the solvent preparation media are used for the robot. In addition, a wash program has been written and instituted to flush all reagent lines, syringes, and flowcells, with copious amounts of wash media after the completion of the automated analysis.

The ideal situation would be to have two identical robotic systems. One unit would be utilized for the development process for automating a method, and the other unit would be dedicated to releasing the product. The program disks could be transferred to the second unit once the validation process and report had been finalized and approved. Currently, Danbury Pharmacal is not in this situation. It makes it quite difficult to time-manage the validation and releasing products. The second automated product method is near completion and the company is now gearing for a third product validation.

### Conclusion

The author's company's experience with robotics, during the past few years, has been a challenging but positive one. With aid from the dedicated staff, and continued support from the management, robotics will succeed and continue to expand into different facets in the organization.

### Acknowledgements

The help of Drew Hund, Group Leader Robotics, Technical Services Department, Danbury Pharmacal, Inc. is gratefully acknowledged.

